# Resolvin D1 promotes the resolution of inflammation in the ACLF rat model by increasing the proportion of Treg cells

**DOI:** 10.1002/iid3.1076

**Published:** 2023-11-20

**Authors:** Linjun Chen, Yixuan Huang, Yizhen Chen, Jiaxuan Chen, Xueye You, Laiyu Zou, Jiabing Chen, Zhixin Chen, Xiaozhong Wang, Yuehong Huang

**Affiliations:** ^1^ Department of Infectious Disease Fujian Medical University Union Hospital Fuzhou China; ^2^ Department of Gastroenterology, Fujian Institute of Digestive Disease Fujian Medical University Union Hospital Fuzhou China; ^3^ Department of Internal Neurology Fujian Medical University Union Hospital Fuzhou China; ^4^ Department of Pathology, The First Affiliated Hospital of Xiamen University, School of Medicine Xiamen University Xiamen China; ^5^ Fujian Clinical Research Center for Digestive System Tumors and Upper Gastrointestinal Diseases Fuzhou China

**Keywords:** acute‐on‐chronic liver failure, anti‐inflammation, IL‐6, rats, resolvin D1, Treg cells

## Abstract

**Objective:**

Acute‐on‐chronic liver failure (ACLF) causes organ system failures in patients and increases the risk of mortality. One of the main predictors of ACLF development in patients is the severity of systemic inflammation. The purpose of this study was to explore the effects of resolvin D1 (RvD1) on the rat model of ACLF.

**Methods:**

The ACLF rats were induced by first intraperitoneally (ip) injecting CCl_4_ and porcine serum for 6 weeks to establish the chronic liver injury, followed by once administration (ip) of lipopolysaccharide and d‐galactose d‐GalN to cause acute liver injury (ALI). An hour before the ALI‐induced treatment, rats were administrated (ip) with 0.9% saline or different doses of RvD1 (0.3 or 1 µg/kg). Afterward, the control and treated rats were killed and samples were collected. Biochemical analysis, hematoxylin‐eosin and Sirius red staining, flow cytometry assay, and real‐time polymerase chain reaction were used to assess the rat liver histopathological injury, the percentage of Treg cells in the spleen, and the messenger RNA (mRNA) levels of transcription factors and immunologic cytokines in liver.

**Results:**

The necroinflammatory scores and the serum levels of transaminase significantly increased in ACLF rats compared with those in control rats. These impaired changes observed in ACLF rats could be attenuated by the administration of a low dose of RvD1 before the induction of ALI, which was associated with the increased proportion of regulatory T cells (Treg) in the spleen together with the increased gene expression ratio of Foxp3/RORγt and decreased mRNA level of *Il‐17a* and *Il‐6* in the liver.

**Conclusion:**

A low dose of RvD1 can promote the resolution of inflammation in ACLF rats by increasing the proportion of Treg cells. RvD1, therefore, may be used as a potential drug for the treatment of patients with ACLF.

## Graphical abstract

Our results showed that low doses of resolvin 1 (RvD1) can prompt inflammation resolution in acute‐on‐chronic liver failures (ACLFs) by increasing the proportion of regulatory T cells (Treg), which may be related to the decrease of interleukin (IL)‐6. We propose a protective Treg‐mediated role for a low dose of RvD1 by which the liver injury of ACLF rats is suppressed and improves survival. These results may help to make clear the pathogenesis of ACLF and find effective therapeutic drugs, which may decrease ACLF mortality and reduce the healthcare burden.

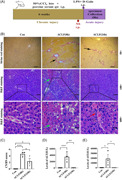

## 1 INTRODUCTION

The term “Acute‐on‐chronic liver failure” (ACLF) was first proposed in 1955, which is used to describe a clinical syndrome with acute decompensation of chronic liver disease or cirrhosis. In patients with cirrhosis and chronic liver disease, ACLF is emerging as a major cause of mortality due to multiple organ dysfunction or severe infection.^1^ Expect for the basic management such as organ support therapies in the clinic, the only known and efficacious treatment for these patients is liver transplantation which, however, can only be applied in a limited number of patients with strict requirements.^2^, ^3^

Inflammation protects the host from the invasion of pathogens. Proper regulation of inflammation may contribute to maintaining homeostasis in the body. On the other hand, aberrant and uncontrolled inflammation can cause great damage to host tissues and it is a key pathological mechanism of many diseases.^4^ Among them, in patients with ACLF, uncontrolled inflammation is one of the main characteristics of the disease, and the severity of systemic inflammation in these patients could be used as a predictor for ACLF progression.^5^

Resolvin D1 (RvD1), produced from the omega‐3 polyunsaturated fatty acid in the body, is one of the specialized proresolving mediators (SPMs). SPMs as a novel family of endogenous lipids have been studied widely due to their significant anti‐inflammatory properties.^4^, ^6^ RvD1 can limit excessive inflammation in different diseases including hemorrhagic shock,^7^ acute focal brain damage,^8^ aortic dissection,^9^ and acute respiratory distress syndrome.^10^ However, the role of RvD1 in patients with ACLF has not yet been reported. Considering the severity of systemic inflammation in patients with ACLF which can cause multiple organ failure, it is interesting to explore if RvD1 can promote the resolution of inflammation in these patients. Therefore, in this study, we aim to explore the anti‐inflammatory effects of RvD1 on ACLF using a rat model and also to investigate the potential underlying mechanisms.

## 2 MATERIALS AND METHODS

### 2.1 Animals

Male Sprague–Dawley rats weighing 180–200 g were purchased from the animal center of Fujian Provincial Center for Disease Control and Prevention. Rats were kept in an environmental temperature‐ and humidity‐controlled room (22–25°C, 40%–70% humidity) with 12‐h light/12‐h dark cycles and with food and water ad libitum. All animal experiments were approved by the Animal Care Committee of Fujian Medical University and carried out in accordance with the Guidelines for Animal Experiments at Fujian Medical University (approval number: 2019–0107).

### 2.2 Experimental design and the rat model of ACLF

To induce ACLF in the rats, after 7 days' stabilization, the rats were first intraperitoneally (ip) injected with 50% carbon tetrachloride (CCl_4_, 1 mL/kg, every Tuesday and Friday) (CN: 61554; Hengxing) which was dissolved in peanut oil, and porcine serum (0.5 mL, every Wednesday) (S9060; Solarbio) for 6 weeks to induce chronic liver injury (CLI).^11^, ^12^ Then, lipopolysaccharide (LPS, 80 μg/kg) (L2880; Sigma‐Aldrich) and d‐galactose (d‐GalN, 0.5 g/kg) (G0500; Sigma‐Aldrich) were administrated ip once to further induce the acute liver injury (ALI).^13^, ^14^

To explore the effects of RvD1 on the rat model with ACLF, in total, 42 rats were used in this study. The rats (*n* = 42) were randomly allocated into four groups: (1) normal control group (Con, *n* = 6): rats were only treated with normal saline; (2) ACLF rat group (ACLF, *n* = 12): rats were treated with normal saline 1 h before the acute hepatic injury; (3) low‐dose RvD1 group (RvD1‐L, *n* = 12): rats were ip injected with RvD1 (10012554; Cayman) 0.3 μg/kg 1 h before the induction of ALI; and (4) high‐dose RvD1 group (RvD1‐H, *n* = 12): rats were ip injected with RvD1 1 μg/kg 1 h before the induction of ALI. Specimens of groups 2–4 were collected at 8 and 24 h after LPS +d‐GalN injection, respectively (*n* = 6). After the indicated treatment time period, rats were killed, and samples of whole blood, liver, and spleen were collected for the subsequent assessment.

### 2.3 Histological assessment

The liver samples were fixed in 10% formalin solution for 24 h, followed by embedding in paraffin and cutting into 10 μm sections. Necrosis of hepatic cells was assessed by hematoxylin‐eosin (H&E) staining, and hepatic fibrosis was assessed by Sirius red staining. The slides were observed with a microscope (Leica DM2500). The confluent necrosis and/or bridging necrosis (CN/BN) scores were calculated according to the histological grading and staging of chronic hepatitis (Ishak's score),^15^, ^16^ and the criteria were shown in Table 1. The histological assessment was performed and evaluated blindly by a professional pathologist.

**Table 1:** Criteria of the confluent necrosis and/or bridging necrosis scores.

Confluent necrosis	Scores
Absent	0
Focal confluent necrosis	1
Zone 3 necrosis in some areas	2
Zone 3 necrosis in most areas	3
Zone 3 necrosis+occasional portal‐central (P‐C) bridging	4
Zone 3 necrosis+multiple P‐C bridging	5
Panacinar or multiacinar necrosis	6

### 2.4 Biochemical analysis

Serum was collected by centrifuging the whole blood at 4000 rpm for 10 min at 4°C. The serum levels of aspartate aminotransferase (AST) and alanine aminotransferase (ALT) were measured by Backman Kurt au5800 automatic biochemical analyzer (Beckman Kurt Co., Ltd.).

### 2.5 Flow cytometry assay

The spleen samples were ground and filtered to obtain the single‐cell suspension. Red blood cell lysis buffer (R1010; Solarbio) was added to lyse the erythrocytes. Cells were first stained with CD4 monoclonal antibody (OX35), APC, eBioscience™ (17‐0040‐80; Thermo Fisher Scientific) for 30 min at 4°C, then the nuclear membrane was broken by Foxp3 transcription factor staining buffer set (00‐5523‐00; Thermo Fisher Scientific), and finally, cells were stained with Foxp3 monoclonal antibody (FJK‐16s), PE, eBioscience™ (12‐5773‐80; Thermo Fisher Scientific) for 30 min at room temperature to mark the Treg cells. The detection of Treg cells was performed using a BD Accuri C6 Plus flow cytometer (BD).

### 2.6 Real‐time polymerase chain reaction (RT‐PCR)

Total RNA of rat liver was extracted by RNA isolation total RNA extraction reagent (R401‐01; Vazyme). The complementary DNA (cDNA) was synthesized by HIScript®Ⅲ RT SuperMix for qPCR (+gDNA wiper) (R323‐01; Vazyme). RT‐PCR analysis of the cDNA was performed using the ABI 7500 Real‐Time PCR system (Life) with Taq Pro Universal SYBR qPCR Master Mix (Q712‐02; Vazyme). The relative messenger RNA (mRNA) expression level of the interested genes was determined using the cycle threshold method ($2^{-∆∆C_{t}}$)^17^ with *Actb* as an internal housekeeping gene. Primer sequences are shown in Table 2.

**Table 2:** Primer sequences for real‐time PCR.

Genes	Forward primer(5′–3′)	Reverse primer(5′–3′)
*Ifng*	GCCATCAGCAACAACATAAGTG	CGCTTCCTTAGGCTAGATTCTG
*Il‐4*	CTTACGGCAACAAGGAACACC	AGACCGCTGACACCTCTACA
*Il‐17a*	GAGTGGACTTCACCCTGTGG	GACATTGGATCGCTGGTGGA
*Foxp3*	ACCTGGAAGAATGCCATCCG	GTTGGCTCCTCTTCTTGCGA
*Rorc*	CAGTGCAATGTGGCCTACTC	AACTTGACAGCATCTCTGGAC
*Tgfb1*	CAACAATTCCTGGCGTTACCT	TGTATTCCGTCTCCTTGGTTCA
*Il‐6*	ACTTCCAGCCAGTTGCCTTCTTG	TGGTCTGTTGTGGGTGGTATCCTC
*Actb*	CGCGAGTACAACCTTCTTGC	CCTTCTGACCCATACCCACC

Abbreviation: PCR, polymerase chain reaction.

### 2.7 Data analysis

All data were analyzed using GraphPad Prism version 8 software. The results are presented as mean ± standard error of the mean. For the comparison among several groups one‐way analysis of variance followed by Holm–Sidak multiple comparison test was used. A *p* < .05 was considered statistically significant.

**Table 3:** Group and mortality of experimental animals.

Group	Total (number)	Death (number)	Death rate (%)
Con	6	0	0
ACLF (8 h)	6	1	16.7
ACLF (24 h)	6	3	50.0
RvD1‐L (8 h)	6	0	0
RvD1‐H (8 h)	6	0	0
RvD1‐L (24 h)	6	2	33.3
RvD1‐H (24 h)	6	1	16.7

*Note*: Criteria for ACLF rats included in this study: obvious inflammation, necrosis, and fibrosis, and a significant increase in serum transaminases. (There were two rats with no obvious inflammation, necrosis, and fibrosis and no significant increase in serum transaminases in ACLF (8 h), and these two rats were therefore considered not to meet ACLF criteria and not included in the further analysis in this study).

Abbreviations: ACLF, acute‐on‐chronic liver failure; Con, control; RvD1, resolvin D1.

## 3 RESULTS

### 3.1 The rat model of ACLF

**Figure 1:**
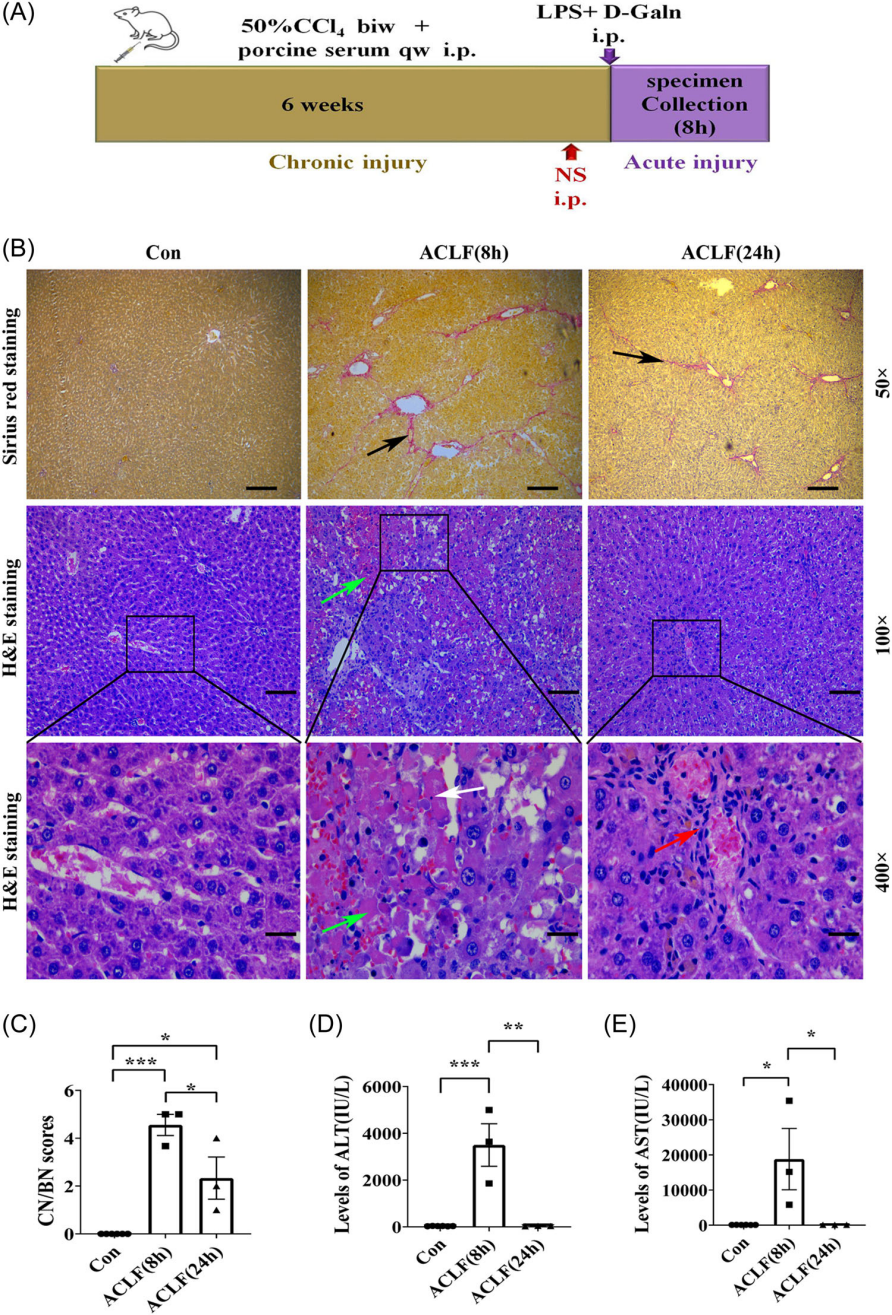
Induction of ACLF in rats. The rat model of the ACLF group was intraperitoneally injected with both CCl_4_ and porcine serum to induce chronic liver injury (CLI), followed by the intraperitoneally treatment of LPS + d‐GalN to induce acute liver injury (ALI). Rats were treated with normal saline 1 h before the administration of LPS + d‐GalN. Samples were collected at 8 or 24 h after the induction of ALI. H&E and Sirius red staining were used to assess rat liver histopathological injury. Serum was collected to examine the levels of transaminase. (A) Schematic of ACLF rat model and experimental protocols; (B) representative staining of the rat liver pathology (black arrow: the proliferation of the collagenous fiber and formation of fibrous septa; green arrow: confluent necrosis; white arrow: apoptosis; red arrow: inflammatory cells infiltration); (C) CN/BN scores of the rat liver pathology; (D) serum ALT levels; and (E) serum AST levels. Data are presented as means ± SEM (*n* = 3–6 rats) (*p < .05; **p < .01; ***p < .001). ACLF, acute‐on‐chronic liver failure; ALT, alanine aminotransferase; AST, aspartate aminotransferase; biw, twice a week; CCl_4_, carbon tetrachloride; CN/BN, the confluent necrosis and/or bridging necrosis; Con, control; d‐GalN, d‐galactose; H&E, hematoxylin and eosin; ip, intraperitoneal injection; LPS, lipopolysaccharide; ns, nonsignificant; qw, once a week; RvD1, resolvin D1.

To induce the pathological changes of ACLF, the rat model of ACLF was induced by subsequent injection of different compounds as illustrated in Figure 1A,^13^, ^14^ and the mortality of rats is show in Table 3. Compared with the healthy rats, the liver capsules of ACLF rats were thickened and there was hard texture adhered to the surrounding tissues. Sirius red staining of liver tissue showed that in the liver of ACLF rats, there was a significant increase in the proliferation of collagenous fiber and the formation of fibrous septa, while healthy rats had no fibrosis (Figure 1B). Next, the CN/BN of hepatic cells was assessed by H&E staining. As shown in Figure 1B, the liver tissue organization of the healthy rats was normal, and their hepatic lobules were well‐arranged. While H&E staining of rat liver pathology showed the disordered structure of hepatic lobule, the swelling, and apoptosis of hepatic cells, inflammatory cells infiltration, and dilated and bleeding hepatic sinus, accompanied by confluent necrosis in ACLF (8 h) rats. The CN/BN scores were significantly higher than the healthy rats (Figure 1C). Though H&E staining of the rat liver pathology in ACLF (8 h) rats also showed the disordered structure of hepatic lobule, in ACLF (24 h) rats, there was a significant decrease in the swelling and apoptosis of the hepatic cells, the inflammatory cells, and the necrosis of the hepatic cells(Figure 1C). Serum transaminases are the classical indicators of hepatocyte inflammation and necrosis. The serum levels of ALT (Figure 1D) and AST (Figure 1E) in ACLF rats were increased substantially at 8 h but showed a dramatic decline at 24 h, which was in line with the previous study.^13^ Thus, the changes in the gross specimens, liver histology, and serological indicators were observed by 8 h but not 24 h after the ALI was induced on the CLI status, which indicated that the rat of the ACLF model was successfully induced at the early time point. Therefore, an early time point (8 h) after ACLF generated in rats was further explored.

### 3.2 The protective effects of RvD1 on ACLF in rat models

**Figure 2:**
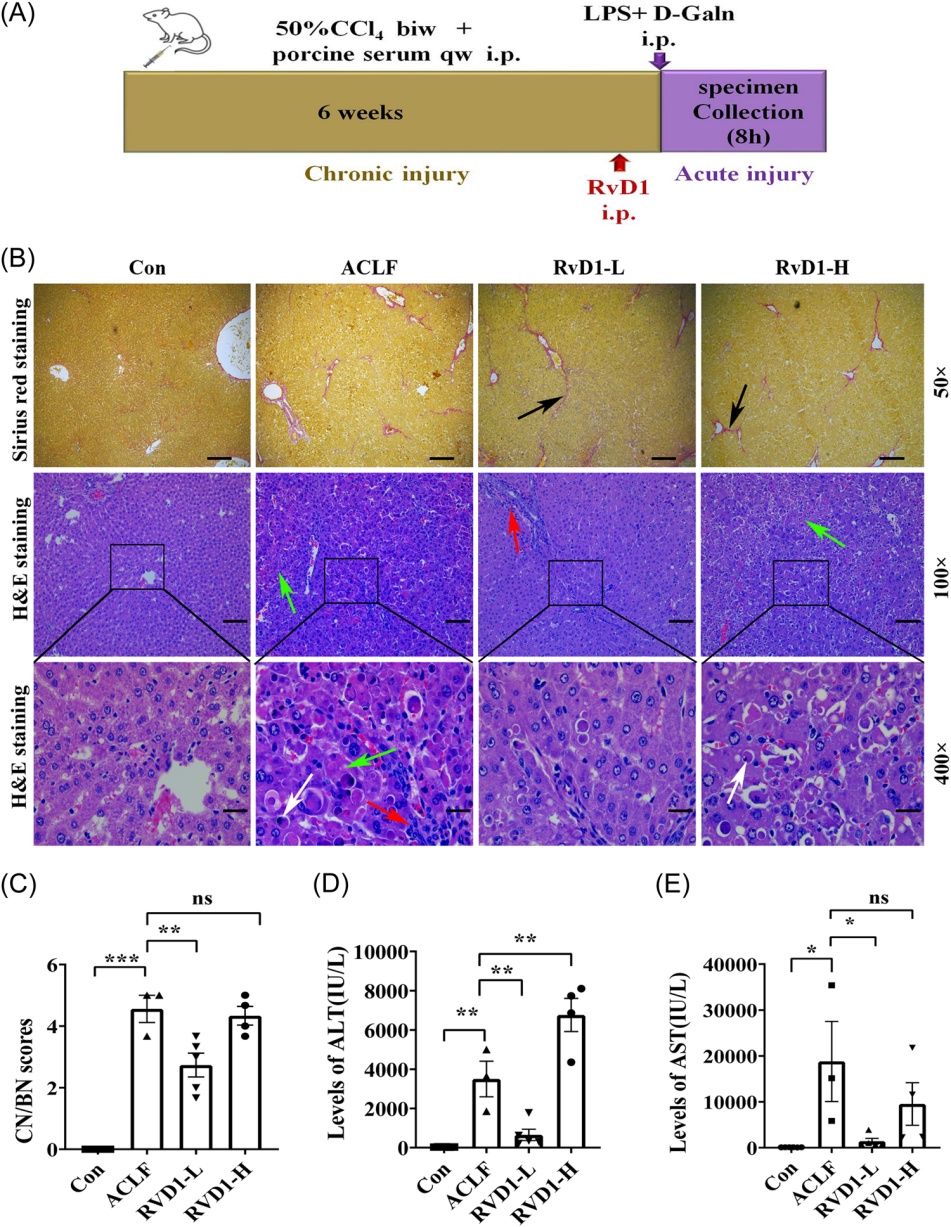
The effects of RvD1 in the rat model of ACLF. The rats of RvD1‐L and RvD1‐H groups were ip injected with both CCl_4_ and porcine serum to induce chronic hepatic injury, and following acute hepatic injury, treated with LPS + d‐GalN ip injection. Rats were treated with RvD1 (0.3 or 1 μg/kg) 1 h before the acute hepatic injury and samples were collected at 8 h after the acute hepatic injury. H&E and Sirius red staining were used to assess rat liver histopathological injury. Serum was collected to examine the levels of transaminase. (A) Schematic of experimental protocols; (B) Sirius red staining and H&E staining of rat liver pathology (black arrow: the proliferation of the collagenous fiber and formation of fibrous septa; green arrow: confluent necrosis; white arrow: apoptosis; red arrow: inflammatory cells infiltration); (C) CN/BN scores; (D) serum ALT levels; and (E) serum AST levels. Data are the mean ± SEM (*n* = 3–6 rats) (*p < .05; **p < .01; ***p < .001). ACLF, acute‐on‐chronic liver failure; ALT, alanine aminotransferase; AST, aspartate aminotransferase; biw, twice a week; CCl_4_, carbon tetrachloride; CN/BN, the confluent necrosis and/or bridging necrosis; Con, control; d‐GalN, d‐galactose; H&E, hematoxylin and eosin; ip, intraperitoneal injection; LPS, lipopolysaccharide; ns, nonsignificant; qw, once a week; RvD1, resolvin D1.

To explore the protective effects of RvD1 in rat models of ACLF, different doses of RvD1 were ip injected 1 h before the induction of ALI in ACI rats (Figure 2A). The Sirius red staining of rat liver pathology showed that compared with the healthy rats, the proliferation of the collagenous fiber and the formation of fibrous septa were significantly increased in the liver of ACLF rats regardless of RvD1 treatment (Figure 2B). H&E staining of rat liver showed that RvD1‐L rather than RvD1‐H administration improved the pathologic changes identified in ACLF rats (Figure 2B). To be specific, the disordered structure of the hepatic lobule, the swelling, and apoptosis of hepatic cells, a little inflammatory cell infiltration, and the inflammatory cells and necrosis of hepatic cells significantly reduced the RvD1‐L group (Figure 2B). H&E staining of rat liver pathology of the RvD1‐H group showed the disordered structure of hepatic lobule, the swelling, and apoptosis of hepatic cells, inflammatory cells infiltration, dilated and bleeding hepatic sinus, accompanied by confluent necrosis (Figure 2B). As shown in Figure 2C, the RvD1‐L group had lower CN/BN scores than the ACLF group. However, the RvD1‐H group still has severe necrosis. In consistence with these pathological changes in the liver, compared with the ACLF rats, a low dose of RvD1 significantly decreased the serum ALT level but a high dose of RvD1 led to an even higher level of ALT (Figure 2D). These findings indicate that a low dose of RvD1 may have a protective effect on liver function in ACLF rats.

Both the anti‐inflammatory effect and the increased ability of hepatocyte regeneration could contribute to the recovery of hepatic pathology in ACLF rats. To evaluate if the liver regeneration in the rat can be affected by the disease of ACLF and/or RvD1 treatment, a marker of cell proliferation (Ki‐67) was assessed in the liver by immunohistochemical staining and the percentage of Ki‐67 positive cells was further calculated. It was shown that in the ACLF group, the percentage of Ki‐67 positive cells was induced by approximately three times compared with the Con group while the treatment of RvD1‐L did not play a role in cell proliferation in the rat model of ACLF (Figure S1A,B).

### 3.3 The effects of RvD1 administration on Treg cells

**Figure 3:**
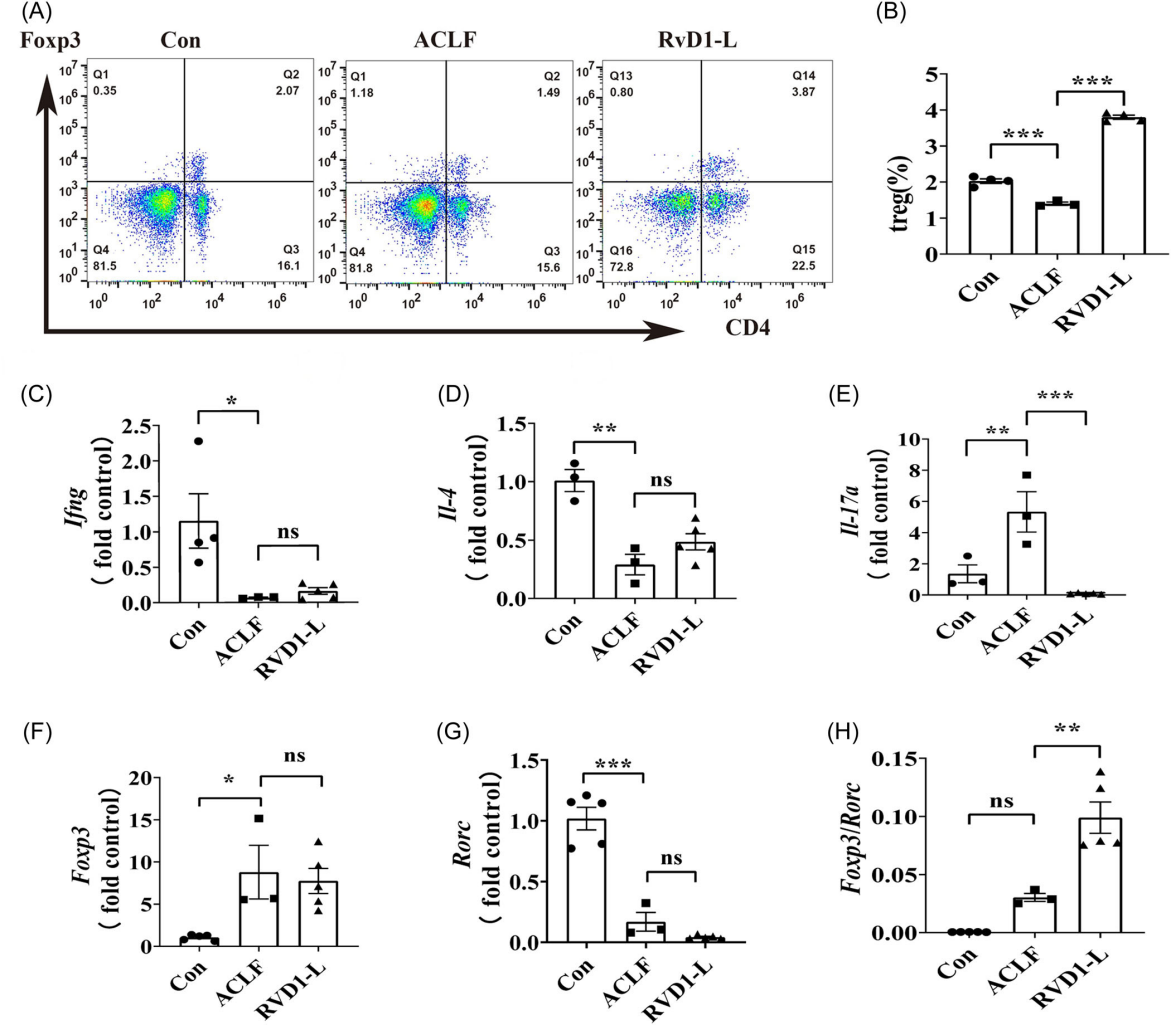
The effects of RvD1 administration on Treg cells in the rat model of ACLF. Flow cytometry assay was used to determine the CD_4_^+^Foxp_3_^+^T cells (Treg cell) population. The mRNA levels of transcription factors and immunologic cytokines in the liver of ACLF rats were examined by real‐time PCR. (A) Representative flow cytometry analysis of CD_4_^+^Foxp_3_^+^T cells obtained from the spleen; (B) percentages of CD_4_^+^Foxp_3_^+^T cells in the spleen; the mRNA expression level in liver as shown in (C) IFNγ, (D) IL‐4, (E) IL‐17, (F) Foxp3, and (G) RORγt; and (H) the mRNA levels of Foxp3/RORγt ratio. Data are the mean ± SEM (*n* = 3–5) (*p < .05; **p < .01; ***p < .001). ACLF, acute‐on‐chronic liver failure; Con, control; IL‐6, interleukin 6; mRNA, messenger RNA; ns, nonsignificant; PCR, polymerase chain reaction; RvD1, resolvin D1; Treg, regulatory T cell.

To explore the potential underlying mechanisms of the protective effects of a low dose of RvD1 on liver function in a rat model of ACLF, Treg cells which have been linked to maintaining immune homeostasis^18^, ^19^, ^20^ were further evaluated. The percentage of Treg cells in the spleen was examined using flow cytometry assays and the results showed that the percentage of Treg cells in the spleen of ACLF rats was lower than that in the healthy rats, which was restored by a low‐dose treatment of RvD1 (Figure 3A,B). The gene expression of the classical downstream cytokines including interferon γ (IFNγ) secreted by T‐helper type 1 (Th1) cells, interleukin (IL)‐4 secreted by Th2 cells, and IL‐17 secreted by Th17 cells were detected by RT‐PCR in the liver tissue. The mRNA levels of *Ifng* and *Il‐4* were significantly increased in the liver of ACLF rats compared with the control rats, while these changes could not be affected by the treatment of RvD1‐L (Figure 3C,D). Furthermore, the increased liver mRNA levels of *Il‐17a* in ACLF rats were significantly attenuated by the treatment of a low dose of RvD1 (Figure 3E). Next, the investigation of the ratio of Foxp3/RORγt (the critical transcription factor of Treg^21^ and Th17,^22^ respectively) which represents a classic way for evaluating the balance of Treg and Th17 cells was further explored in the liver in this study. No significant difference was identified in the changes in *Foxp3* and in *Rorc* between the ACLF group and the RvD1‐L group (Figure 3F,G). However, the ratio of *Foxp3/Rorc* in mRNA levels was higher in the RvD1‐L group than that in the ACLF group (Figure 3H). These results indicate that RvD1 treatment could increase the proportion of Treg cells in the spleen of ACLF rats and regulate the balance of Treg and Th17 in the liver.

**Figure 4:**
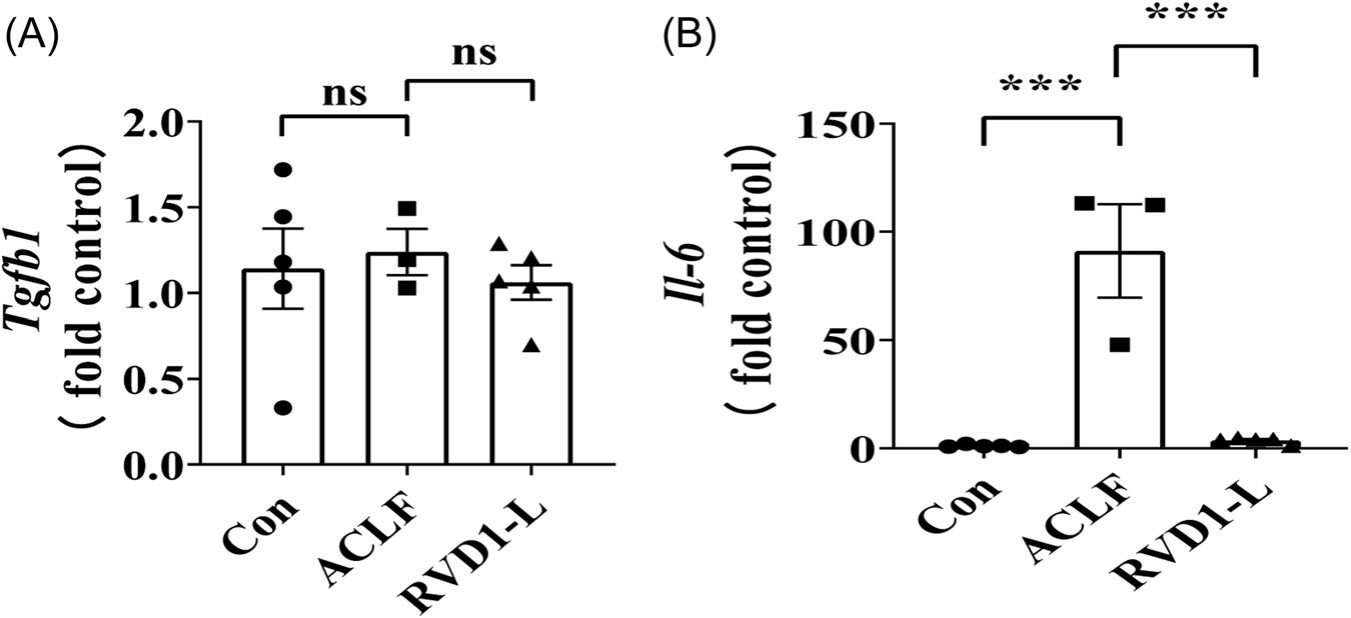
The regulatory effect of RvD1 on the expression of immunologic cytokines in the rat model of ACLF. The mRNA levels of immunologic cytokines in the ACLF rat liver were examined by real‐time PCR. (A) TGF‐β, and (B) IL‐6. Data are the mean ± SEM (*n* = 3–5 rats) (***p < .001). ACLF, acute‐on‐chronic liver failure; Con, control; IL‐6, interleukin 6; ns, nonsignificant; PCR, polymerase chain reaction; RvD1, resolvin D1; TGF‐β, transforming growth factor‐β.

### 3.4 The effects of RvD1 on immunologic cytokines in the liver microenvironment

To explore the possible mechanisms underlying the increased proportion of Treg cells in the liver of ACLF rats treated with RvD1‐L, we further determined the immunologic cytokines (e.g., transforming growth factor‐β [TGF‐β] and IL‐6) as they are closely related to the Treg cell differentiation in the liver microenvironment.^23^, ^24^, ^25^, ^26^ Surprisingly, compared with the Con group, the mRNA expression of *Tgfb1* was not affected in the liver of ACLF rats regardless of the treatment of RvD1 (Figure 4A). However, the mRNA levels of *Il‐6* were significantly elevated in the liver of ACLF rats compared with the control rats, but this increase in the *Il‐6* gene expression restored to a control level by a low‐dose treatment of RvD1 in ACLF rats (Figure 4B).

**Figure 5:**
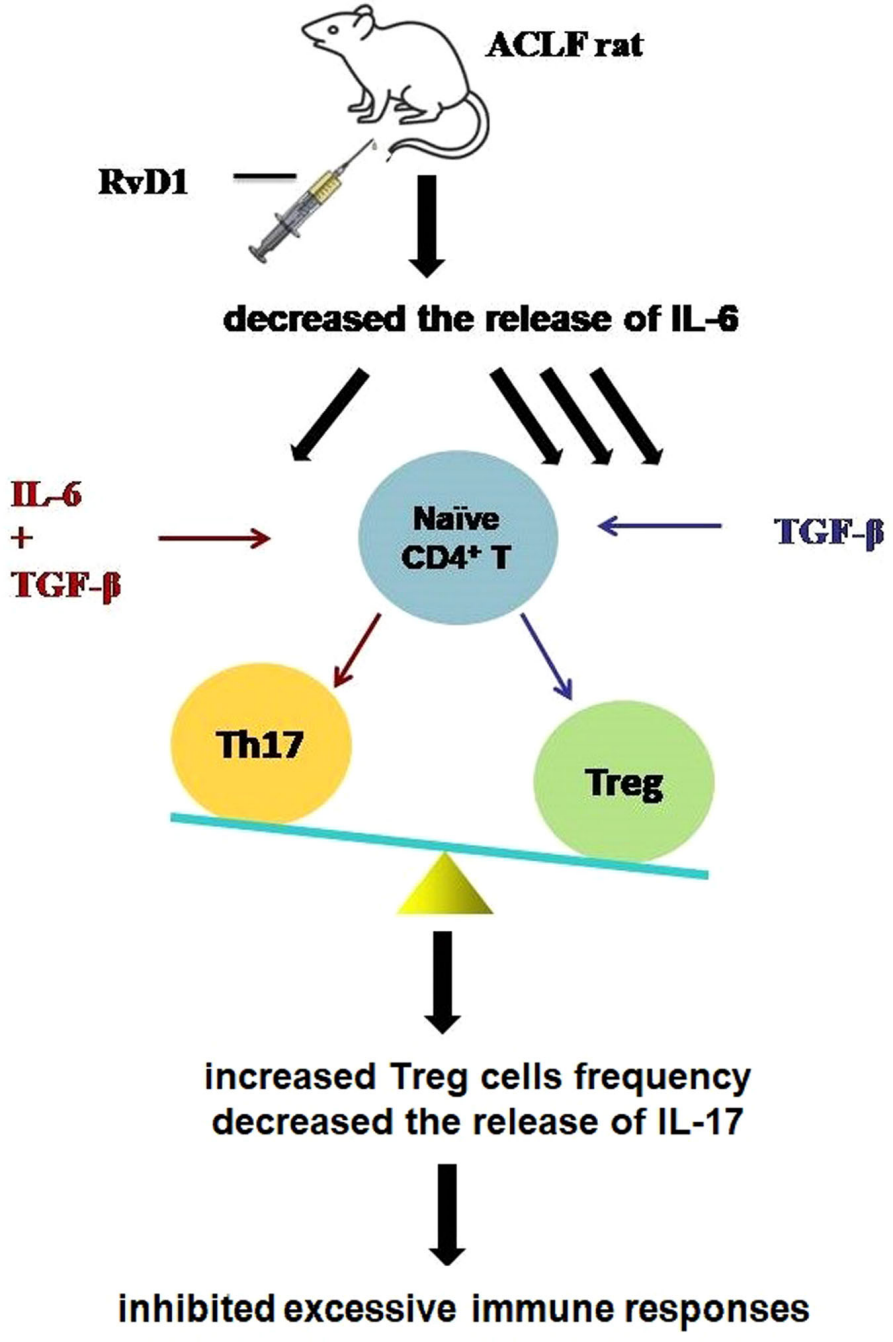
The possible mechanism of resolving the inflammation in ACLF rats of RvD1. A low dose of RvD1 can play a protective role in ACLF rats by increasing the proportion of Treg and decreasing the expression of IL‐6. Treg and Th17 share a common progenitor—naïve CD_4_^+^ T cells and a signaling pathway mediated by TGF‐β. In the absence of IL‐6, TGF‐β drives naïve CD_4_^+^ T cells to differentiate into Treg cells but not Th17 cells, thereby the percentage of the Treg cells is increased, and further decreasing the downstream inflammation cytokine IL‐17 (secreted by Th17 cells). By this mechanism, RvD1 promotes the resolution of inflammation in the liver of ACLF rats. ACLF, acute‐on‐chronic liver failure; IL‐6, interleukin 6; RvD1, resolvin D1; Th17, T‐helper type 17; TGF‐β, transforming growth factor‐β; Treg, regulatory T cell.

## 4 DISCUSSION

In this study, we have identified the protective role of RvD1 in promoting the resolution of inflammation in the rat model of ACLF. The disease progression of ALI induced on rat CLI model could be achieved by 8 h in terms of the obviously identified severe inflammatory and necrosis in the liver and the disrupted liver function. These impaired changes observed in ACLF rats could be attenuated by the preadministration of a low dose of RvD1 before the induction of ALI, which was associated with the increased proportion of Treg cells in the spleen together with the increased gene expression ratio of Foxp3/RORγt and decreased mRNA level of *Il‐17a* and *Il‐6* in the liver.

ACLF refers to the clinical syndrome of acute decompensation of liver function in a short period of time on the basis of chronic liver disease.^5^ A rat model of ACLF was induced to study the effect of RvD1 on this disease with a focus on liver function and the resolution of inflammation in vivo. Two steps were carried out to induce the ACLF in rats and this method has also been studied widely.^13^, ^14^ The CCl_4_ was reported to induce liver lesion and liver fibrosis^27^, ^28^ and at the same time period porcine serum as a xenogeneic protein was given to cause immune damage.^29^ Then, the induction of ALI in the CLI rat was achieved by injection once with LPS and d‐GalN. LPS is the main component of Gram‐negative bacteria and is one of the most potent stimulators of the host's innate immune system.^30^ It has been implicated as an important cofactor in the pathogenesis of the ALI.^31^ d‐GalN can induce liver injury by interfering with the uridine pool in the cell and thus can further disrupt the cell metabolism.^32^ In this study, two different time points after the injection of LPS and d‐GalN in CLI rats were explored and after 8 h, we confirmed the expected disrupted changes in the liver pathology and the liver function, which was in consistence with the changes identified in patients with ACLF.^16^, ^33^

Immune dysregulation, which can lead to excessive immune activation and cytokine storms, is reported to contribute to the development and poor prognosis of ACLF.^34^, ^35^ It is known that Treg cells and Th17 cells share a common progenitor, that is the Naïve CD_4_^+^ T cells, and the direction of their differentiation is determined by the proinflammatory cytokine, namely IL‐6, via a signaling pathway mediated by TGF‐β.^34^, ^35^ In a healthy body, the proper balance of the Treg/Th17 cell ratio is essential. The imbalance of the Treg/Th17 ratio in peripheral blood was previously reported in patients with ACLF.^36^ RvD1, a well‐established anti‐inflammatory molecule, can improve the balance of Treg/Th17 cell ratio in systemic lupus erythematosus^37^ and can alleviate renal tubular injury by increasing the percentage of Treg cells. In the current study, for the first time, we studied the effect of RvD1 on immunoregulation in ACLF in vivo. Our data showed that compared with the healthy rats, ACLF rats had significantly decreased proportion of Treg cells in the spleen and increased levels of proinflammatory cytokines (e.g., IL‐17 and IL‐6) in the liver, which was in consistence with the previous findings.^38^, ^39^ These altered changes in ACLF rats were restored by a low dose of RvD1. In the presence of TGF‐β and IL‐6, the Naïve CD_4_^+^ T cells can differentiate into Th17 cells which mainly secrete IL‐17 to play a proinflammatory role. Therefore, it is possible that RvD1 plays a role in reducing IL‐6 gene expression in the liver of ACLF rats resulting in a lower level of liver gene expression of IL‐17. The accentuated ratio of *Foxp3/Rorc* by a low dose of RvD1 may also contribute to the promoted resolution of inflammation in RvD1‐treated rats with ACLF (Figure 5). Moreover, the Naïve CD_4_^+^ T cells can also differentiate into Th1 cells secreting IFNγ and Th2 cells secreting IL‐4, respectively. The reduced gene expression level of *Ifng* and *Il‐4* in the liver of ACLF rats in this study could not be improved by the treatment of RvD1. In the present study, however, we cannot clarify if the Treg cells identified in the liver are differentiated from the naïve t‐cells in the liver or from the differentiated Treg cells in the circulation infiltrating into the liver, which needs to be further investigated.

In summary, our study indicates that a low dose of RvD1 can promote the resolution of inflammation in ACLF rats by increasing the proportion of Treg cells. Thus, the application of a proper dose of RvD1 could provide a new preventive strategy for CLI patients with a high risk to develop into ACLF. However, the effects of RvD1 on different types of liver failure other than the current model of drug‐induced ACLF need to be further investigated.

## AUTHOR CONTRIBUTIONS

**Linjun Chen**: Conceptualization; data curation; funding acquisition; methodology; writing—original draft. **Yixuan Huang**: Investigation; validation. **Yizhen Chen**: Investigation. **Jiaxuan Chen**: Formal analysis; validation. **Xueye You**: Investigation. **Laiyu Zou**: Resources; validation. **Jiabing Chen**: Investigation. **Zhixin Chen**: Resources. **Xiaozhong Wang**: Project administration; supervision. **Yuehong Huang**: Conceptualization; funding acquisition; methodology; supervision; writing—review and editing.

## CONFLICT OF INTEREST STATEMENT

The authors declare no conflict of interest.

## Supporting information

Supporting information.

## Data Availability

The datasets generated during and/or analyzed during the current study are available from the corresponding author upon reasonable request.
