# Supplementary material for: Resolvin D1 promotes the resolution of inflammation in the ACLF rat model by increasing the proportion of Treg cells
Source: Immun Inflamm Dis. 2023 Nov 20;11(11):e1076. doi: 10.1002/iid3.1076 (PMC10659757; doi:10.1002/iid3.1076)
Supplement: Supplementary file 1 — Supporting information. [file IID3-11-e1076-s001.docx]

# Supplementary material


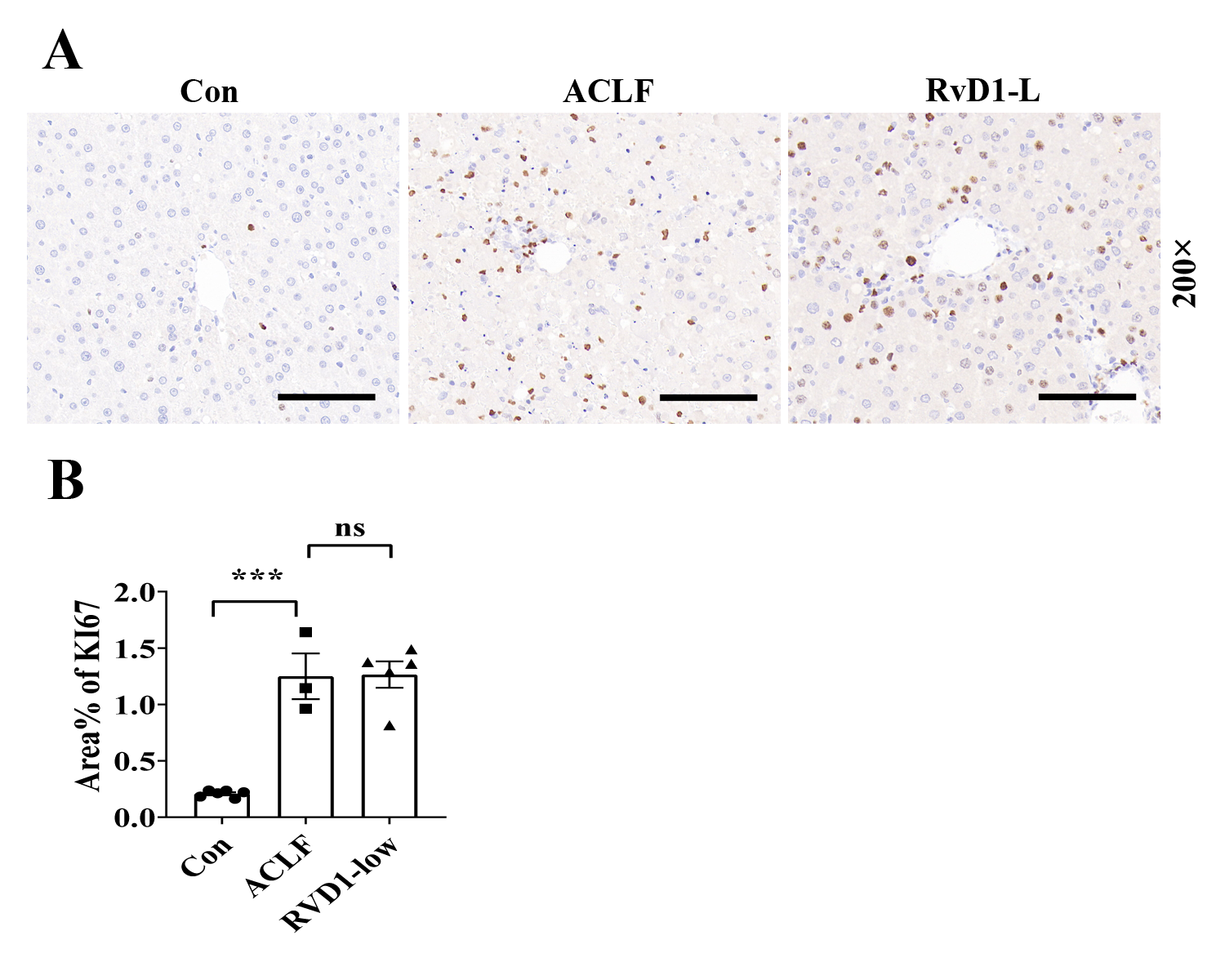


# Figure legends

[**Supplement**](javascript:;)**ary Figure 1.Immunohistochemistry (IHC) of Ki-67 in liver.**

IHC was perfomed by using rabbit anti-Ki-67 antibody (1:1000, GB111141, Servicebio, Wuhan, China) as the primary antibody and HRP- labeled goat anti-rabbit secondary antibody (1:200, GB23303, Servicebio, Wuhan, China). (A) Representative image of

IHC staining of Ki67 (Positive expression was indicated in brown); (B) The percentage of Ki67 postive cells was calculated and express as Area% of Ki67. n=3-6 rats. One-way ANOVA followed by Holm–Sidak multiple comparison test was used.,**P*<0.05, ***P*<0.01, ****P*<0.001.
